# Supplementary material for: Gate tunable light–matter interaction in natural biaxial hyperbolic van der Waals heterostructures
Source: Nanophotonics. 2022 Apr 4;11(10):2329–40. doi: 10.1515/nanoph-2022-0034 (PMC11636507; doi:10.1515/nanoph-2022-0034)
Supplement: Supplementary file 1 — Supplementary Material [file j_nanoph-2022-0034_suppl.pdf]

Supplementary Information - Gate tunable light-matter interaction in natural biaxial hyperbolic van der Waals heterostructures

Aneesh Bapat,[1] Saurabh Dixit,[1] Yashika Gupta,[1] Tony Low,[2] and Anshuman Kumar[1,*]

[1] *Laboratory of Optics of Quantum Materials, Physics Department, Indian Institute of Technology Bombay, Mumbai - 400076, India*

[2] *Department of Electrical and Computer Engineering, University of Minnesota, Minneapolis, Minnesota 55455, USA*

(Dated: January 26, 2022)

* anshuman.kumar@iitb.ac.in

S1. MATERIAL PROPERTIES OF α-MOO$_3$ AND GRAPHENE

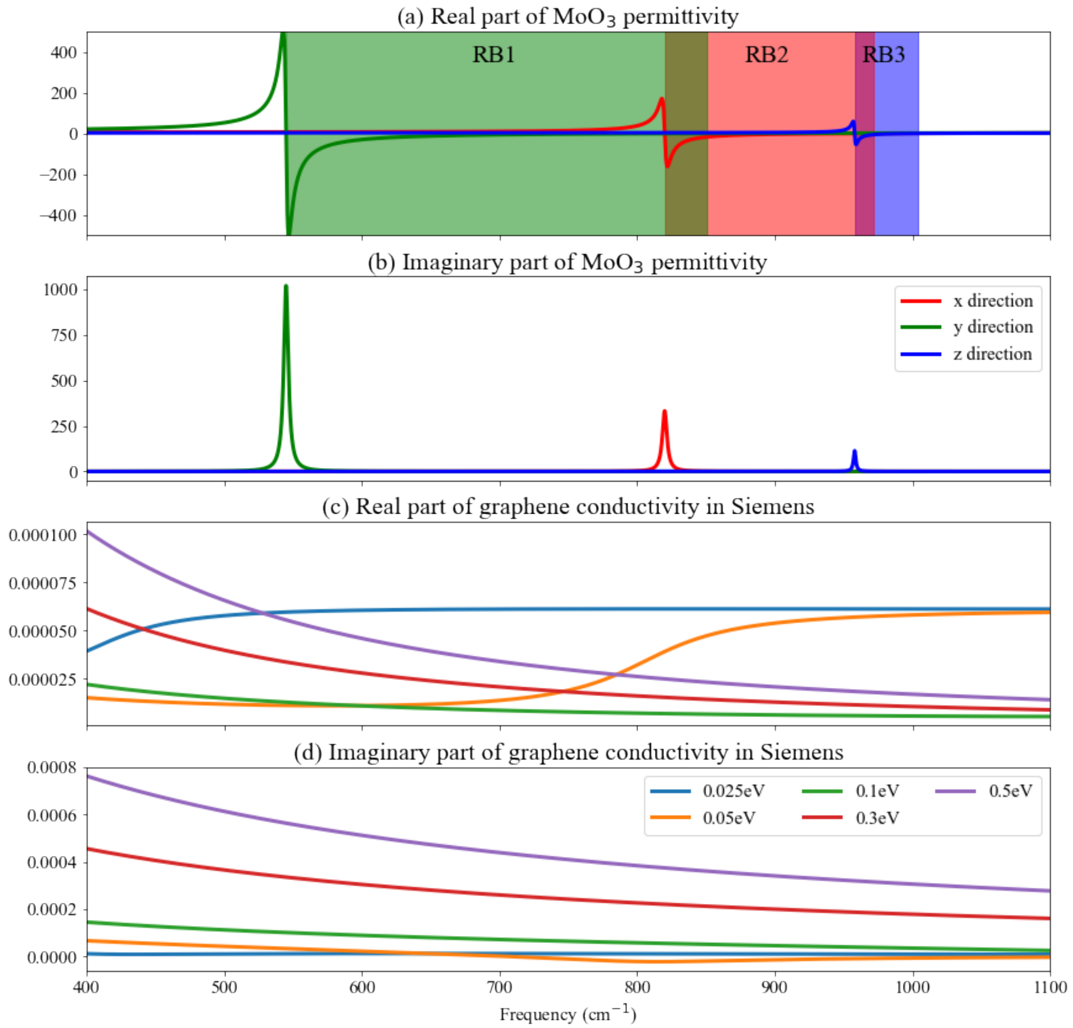

FIG. S1. (a) and (b) represent real and imaginary part of relative permittivity of α-MoO$_3$ in the $x-$, $y-$ and $z-$ crystal direction demonstrating three RB spectral regions. (c) and (d) represent real and imaginary part of conductivity of graphene in Siemens with varying chemical potential.

S2. EFFECT OF THICKNESS OF α-MOO$_3$ ON HPPP DISPERSION

We present here two representative thicknesses of α-MoO$_3$, mainly 100nm and 200nm, to examine the effect of thickness on dispersion of HPPP modes as a function of chemical potential of graphene and ϕ. For no graphene case, we observe relatively stronger oscillator strength for higher-order hyperbolic PhP modes in the dispersion curve, shown in Figs. S2(a)-(c) and S3(a-c), compared to smaller thickness (50nm). Energy spacing between two higher-order PhP modes decreases with increasing thickness of α-MoO$_3$ which is expected from the particle in a box analogy. Sharp increase in the density of phononic states can be observed with the increase in the thickness of α-MoO$_3$. However, the dispersion of HPPP modes for all thicknesses follows almost similar trends, as shown in Fig. 2 of main text, and Figs. S2 and S3, for various chemical potential values.

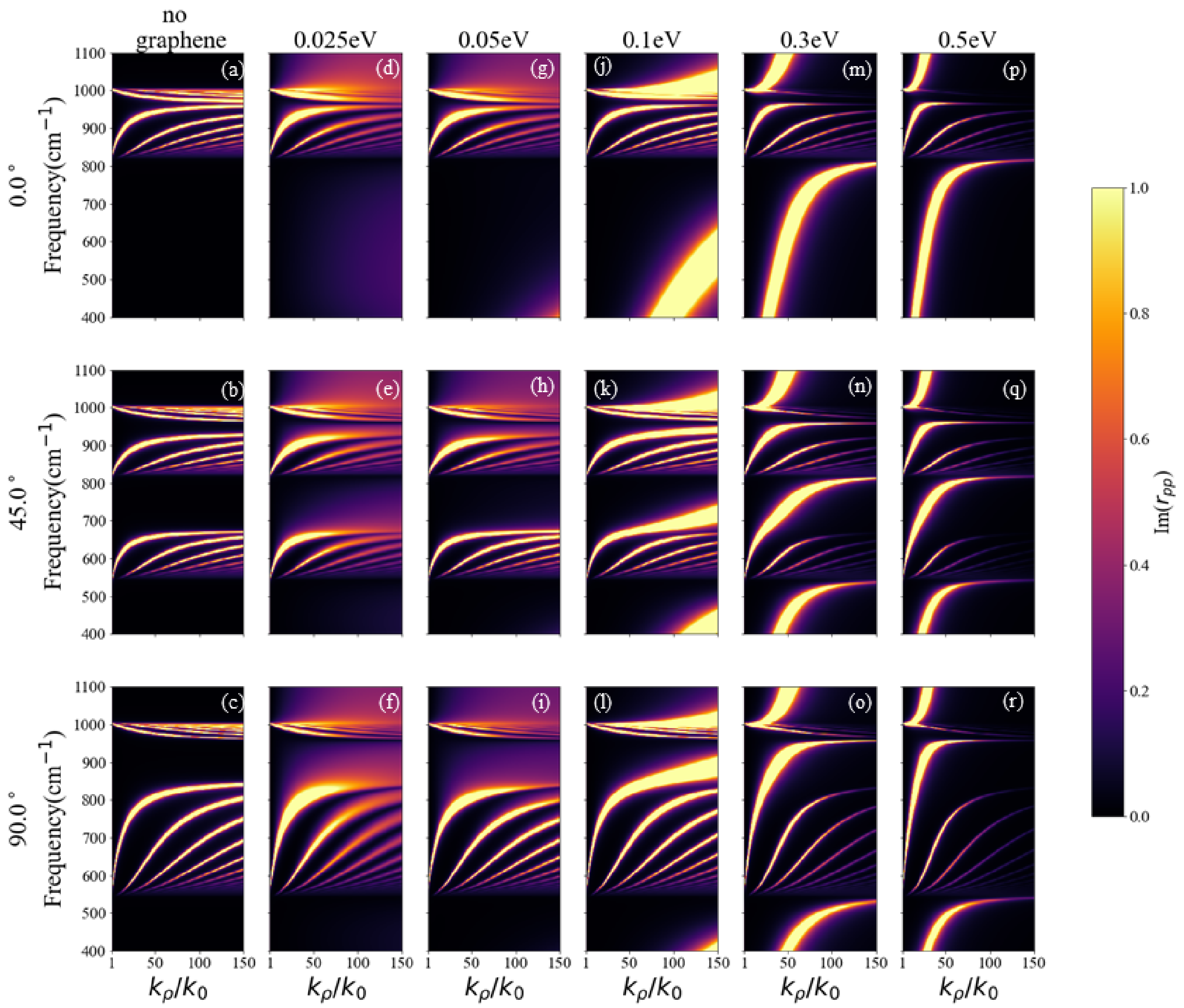

FIG. S2. HPPPs dispersion as a function of chemical potential and ϕ for 100nm thin film of α-MoO_3

S3. ISO-FREQUENCY SURFACES FOR HPPPS OF GRAPHENE AND α-MOO$_3$ HETEROSTRUCTURE

We investigate the imaginary part of r_{pp} at the representative frequencies (ω) for the RB-1, RB-2, transition between RB-2 and RB-3, and RB-3 spectral region as function of mode index and ϕ demonstrated by radial and azimuthal direction of polar plots. Hybridization of hyperbolic PhPs and SPPs can be seen with increasing chemical potentials (μ) above $0.1eV$ as shown in figure S4. Iso-frquency surfaces of these four frequencies for chemical potential above $0.1eV$ are combined and represented as Fig.3 in the main paper.

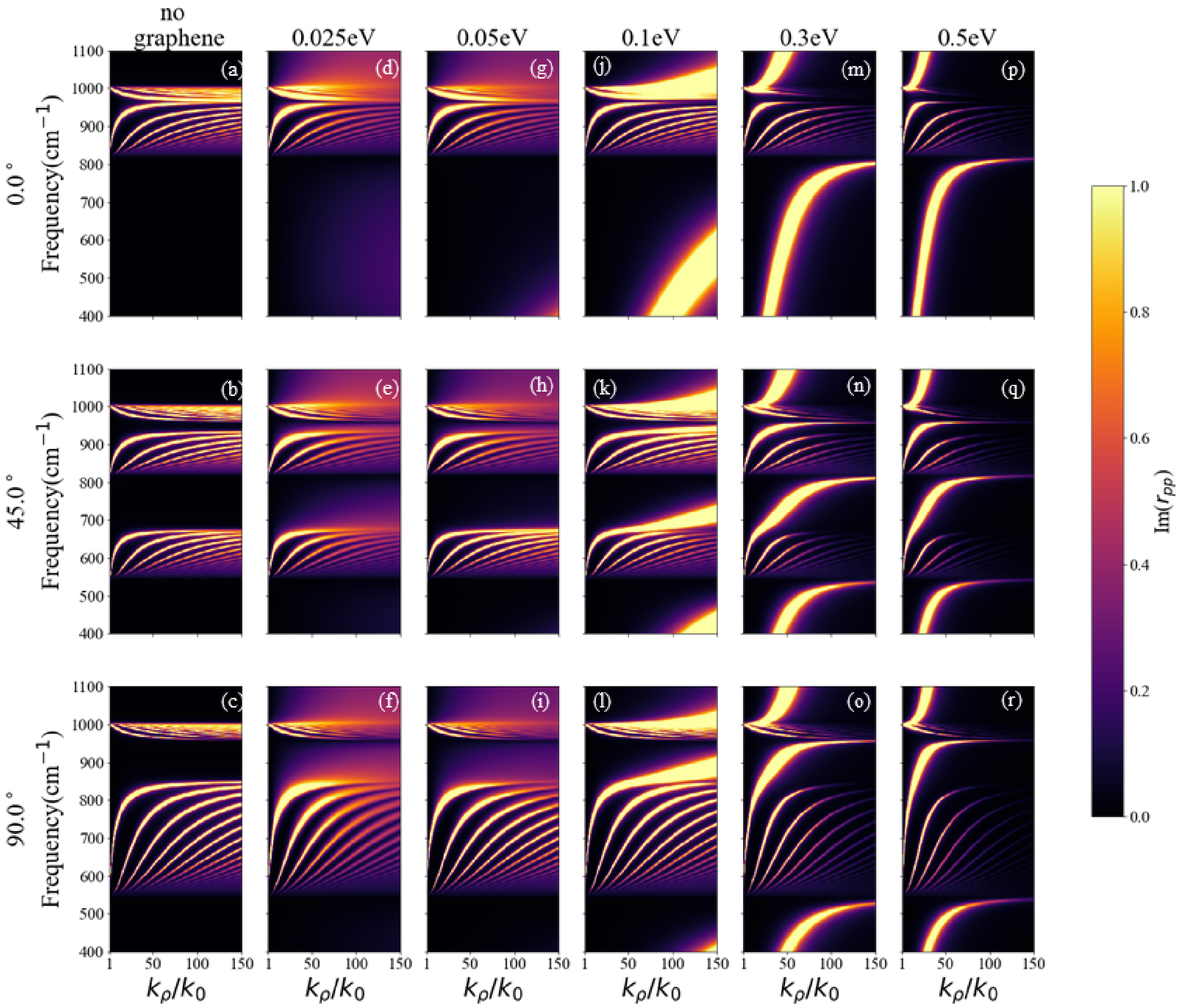

FIG. S3. HPPPs dispersion as a function of chemical potential and ϕ for 200nm thin film of α-MoO_3

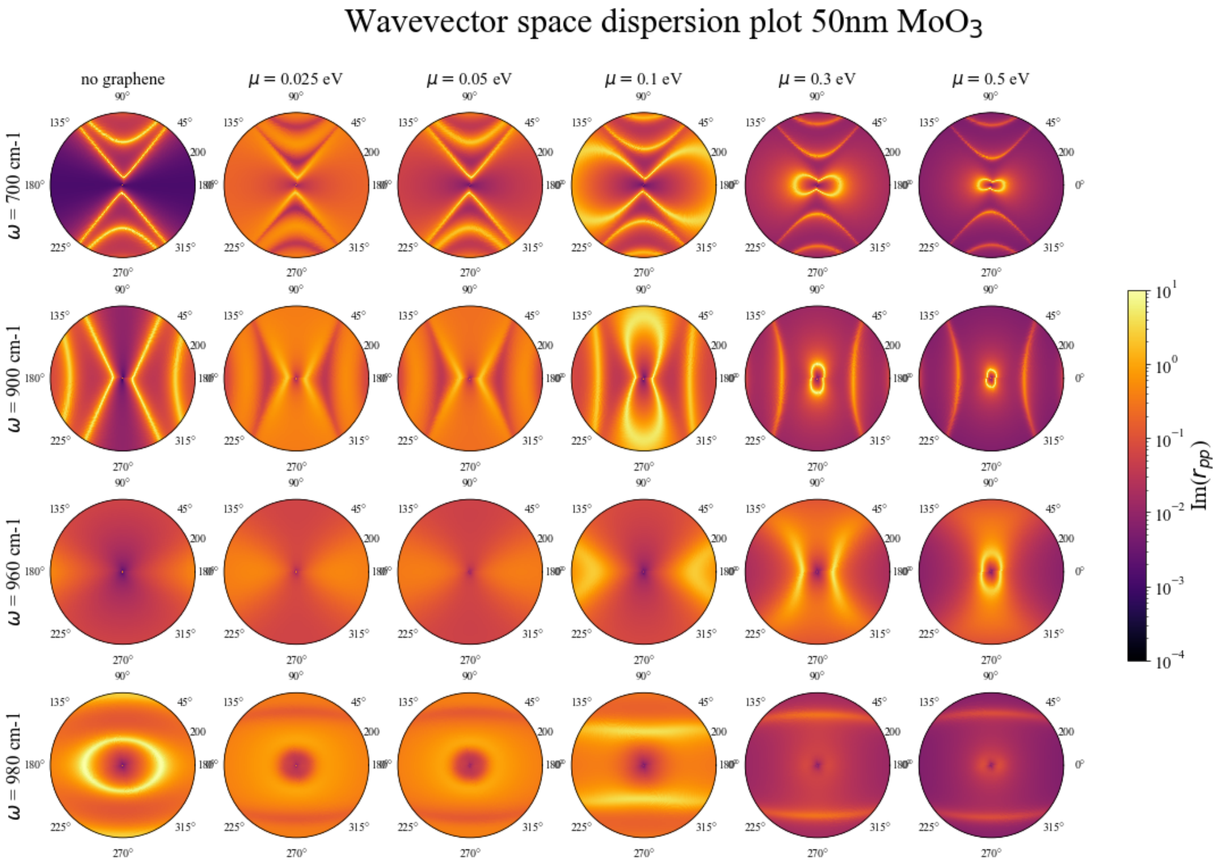

FIG. S4. Iso-frequency polar plots for $\omega = 700\text{cm}^{-1}$, 900cm^{-1},960cm^{-1} and 980cm^{-1} and chemical potential $(\mu) = 0.025eV$, $0.05eV$, $0.1eV$, $0.3eV$, and $0.5eV$ to examine hybrid plasmon phonon polariton modes.

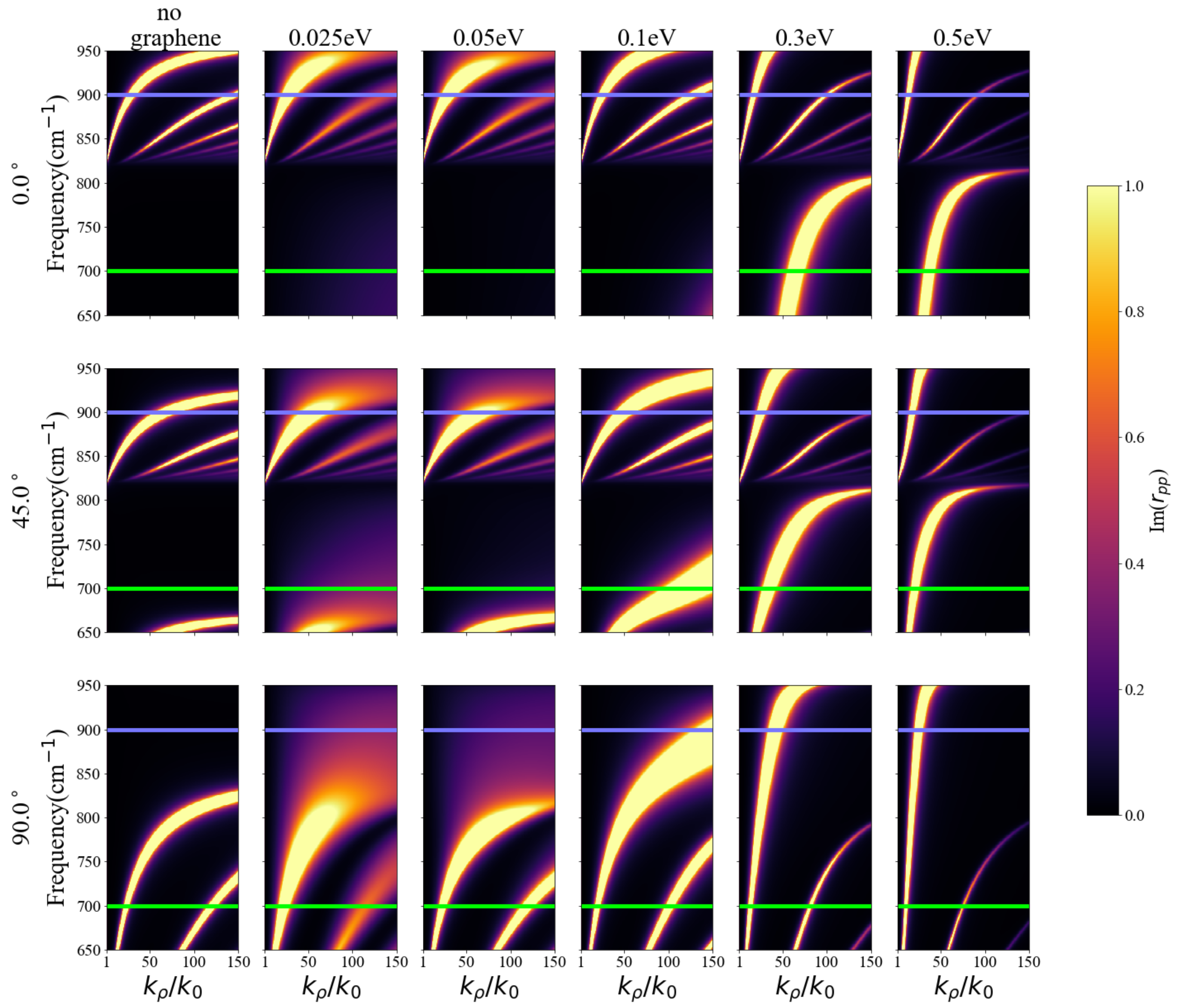

FIG. S5. HPPPs dispersion as a function of chemical potential and ϕ for 50nm thin film of α-MoO_3 in the spectral region 650 cm^{-1} to 950 cm^{-1}.

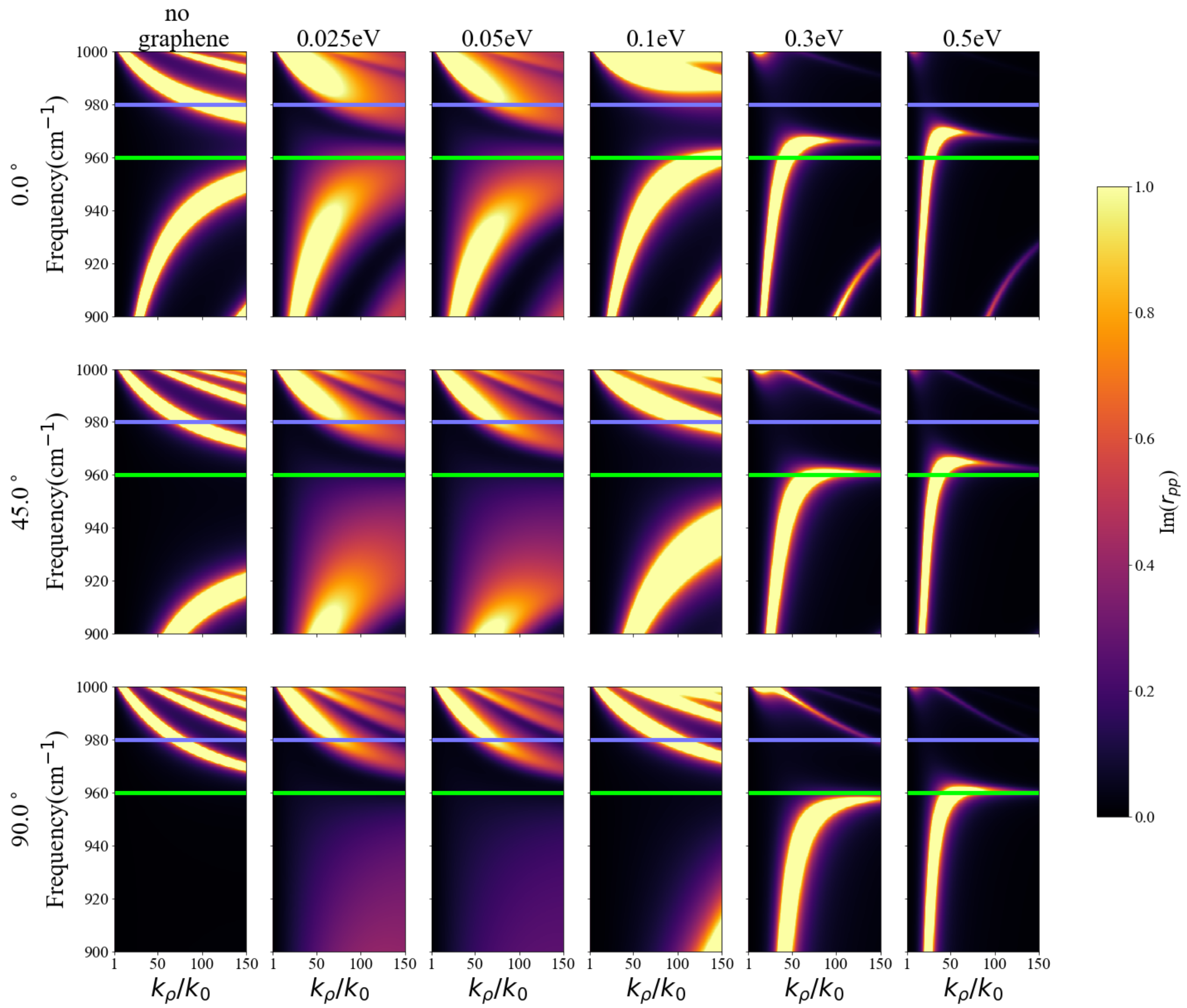

FIG. S6. HPPPs dispersion as a function of chemical potential and ϕ for 50nm thin film of α-MoO_3 in the spectral region 900 cm^{-1} to 1020 cm^{-1}.

S4. EFFECT OF THICKNESS OF α-MOO$_3$ ON ANISOTROPIC SPONTANEOUS EMISSION RATES

A. Anisotropic SERs of α-MoO$_3$ without graphene

We examine the effect of thickness of α-MoO$_3$ without graphene on anisotropic SERs. The SERs for MoO$_3$ thickness of 50nm,100nm and 200nm (without graphene) are plotted in the Fig. S7 for $x-$, $y-$, and $z-$ polarized electric dipole. For $z_\circ = 50$nm, SER spectra for $x-$ ($y-$) polarized electric dipole exhibits broadband SER with the order of magnitude around 10^3 in the RB-2 (RB-1) spectral region, which is due to biaxial hyperbolic anisotropy of α-MoO$_3$. However, for $z_\circ = 200$nm, we observe a reduction of SER by one order of magnitude due to the evanescent nature of hyperbolic PhP modes, resulting in less interaction of polaritonic modes with electric dipole and hence reduction of SER. We found that the order of anisotropic SERs does not change significantly with thickness of α-MoO$_3$. Furthermore, line-width and oscillator strength of spectral dips decreases with increasing thickness. As we increase the thickness of α-MoO$_3$, increases the number of modes available at smaller k_ρ, resulting in the enhancement of SERs. This can also be seen from dispersion plots in the Figs.S2 and S3. Therefore, the strength of spectral dip decreases due to the modulation of the phononic density of states with the thickness of α-MoO$_3$. However, at $z_\circ = 200nm$, modes at higher k_ρ are evanescent and die out exponentially with height, and thus, unavailable to the dipole. Hence, line-width of spectral dip is more prominent at $z_\circ = 200nm$.

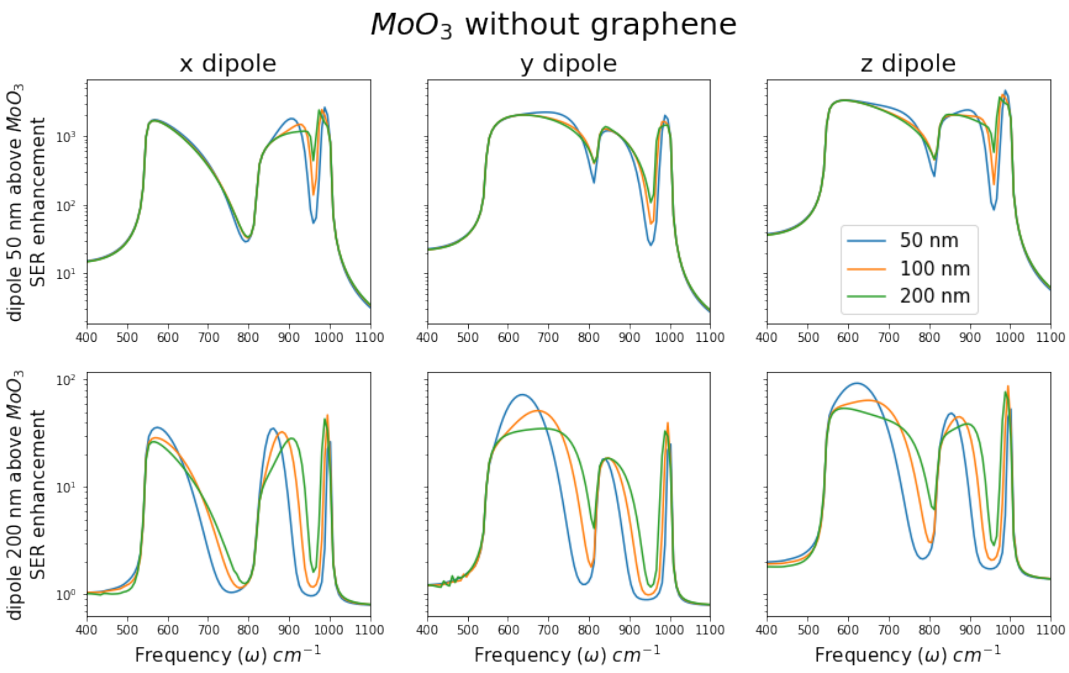

FIG. S7. Anisotropic SERs from α-MoO$_3$: (a)-(c) represent SERs of $x-$, $y-$ and $z-$ polarized electric dipole, respectively, at $z_\circ = 50$nm for different thicknesses. (d)-(f) represent SERs of $x-$, $y-$ and $z-$ polarized electric dipole, respectively, at $z_\circ = 200$nm for different thicknesses.

B. Anisotropic SERs of α-MoO$_3$ and interband optical transition dominated graphene

Figure S8 presents anisotropic SERs for $\mu = 0.025eV$ and $0.05eV$ and α-MoO$_3$ without graphene. The anisotropic SERs for $\mu = 0.025eV$ and $0.05eV$ are found nearly similar to no graphene case in the RB spectral regions due to absence of HPPP formations. Furthermore, a significant difference between the spectral dips are observed in no graphene and with graphene cases due to interband optical transitions of graphene which makes phonon polariton modes lossy. Graphene/α-MoO$_3$ heterostructure exhibit remarkably large anisotropic SERs outside the RB spectral regions, which is associated with the SPP modes of graphene. Next, we examine effect of thickness of α-MoO$_3$ on anisotropic SERs from proposed heterostructure. Figures S9 and S10 correspond to anisotropic SERs for α-MoO$_3$ thickness of 100nm and 200nm respectively. The order of SERs, at $z_\circ = 50$nm and 200nm, is found nearly similar for all the thicknesses. Oscillator strength and line-width of the spectral dips decreases with increasing thickness of α-MoO$_3$, which is, as discussed above, due to

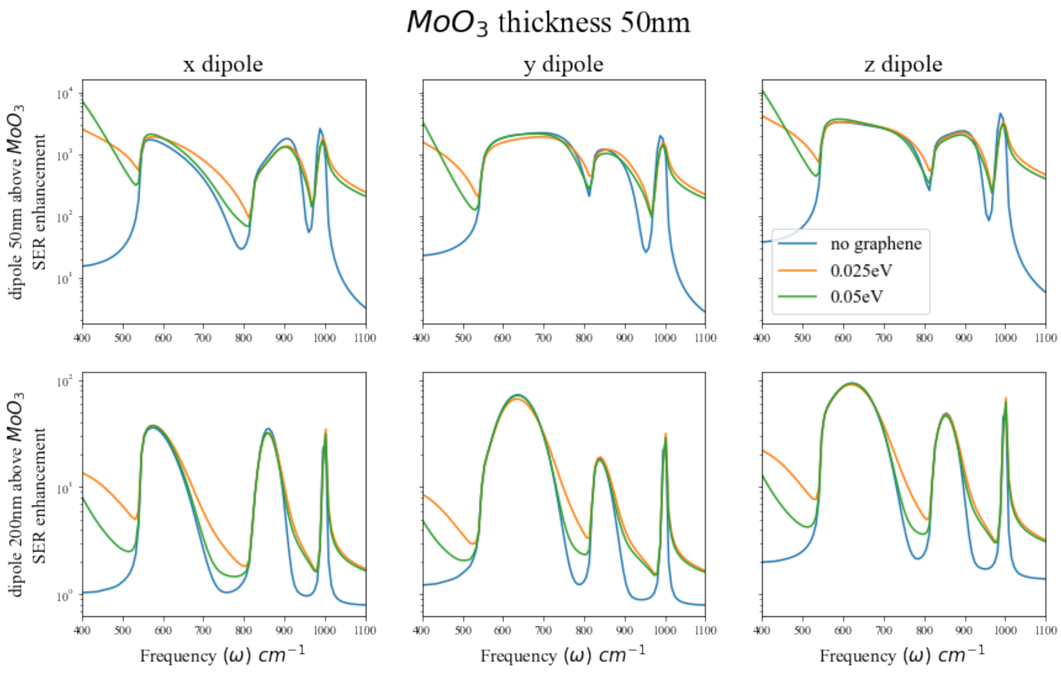

FIG. S8. Anisotropic SERs from a 50nm thin film of α-MoO_3: (a)-(c) represent SERs of $x-$, $y-$ and $z-$ polarized electric dipole, respectively, at $z_\circ = 50$nm for different μ values. (d)-(f) represent SERs of $x-$, $y-$ and $z-$ polarized electric dipole, respectively, at $z_\circ = 200$nm for different μ values.

modulation of phononic density of states of α-MoO_3.

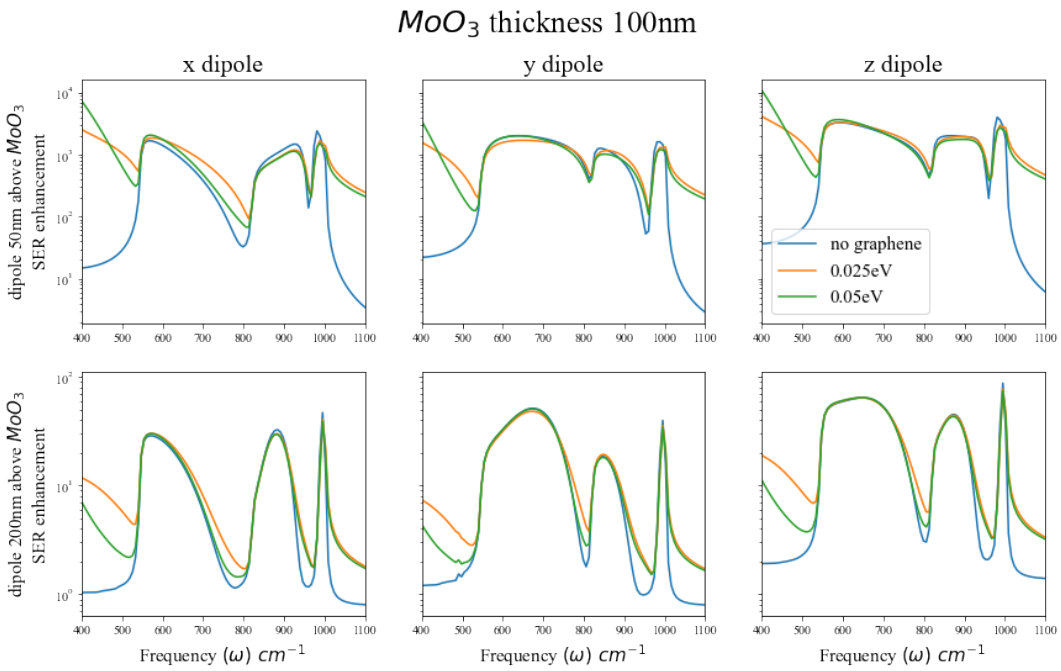

FIG. S9. Anisotropic SERs from a 100nm thin film of α-MoO_3: (a)-(c) represent SERs of $x-$, $y-$ and $z-$ polarized electric dipole, respectively, at $z_\circ = 50$nm for different μ values. (d)-(f) represent SERs of $x-$, $y-$ and $z-$ polarized electric dipole, respectively, at $z_\circ = 200$nm for different μ values.

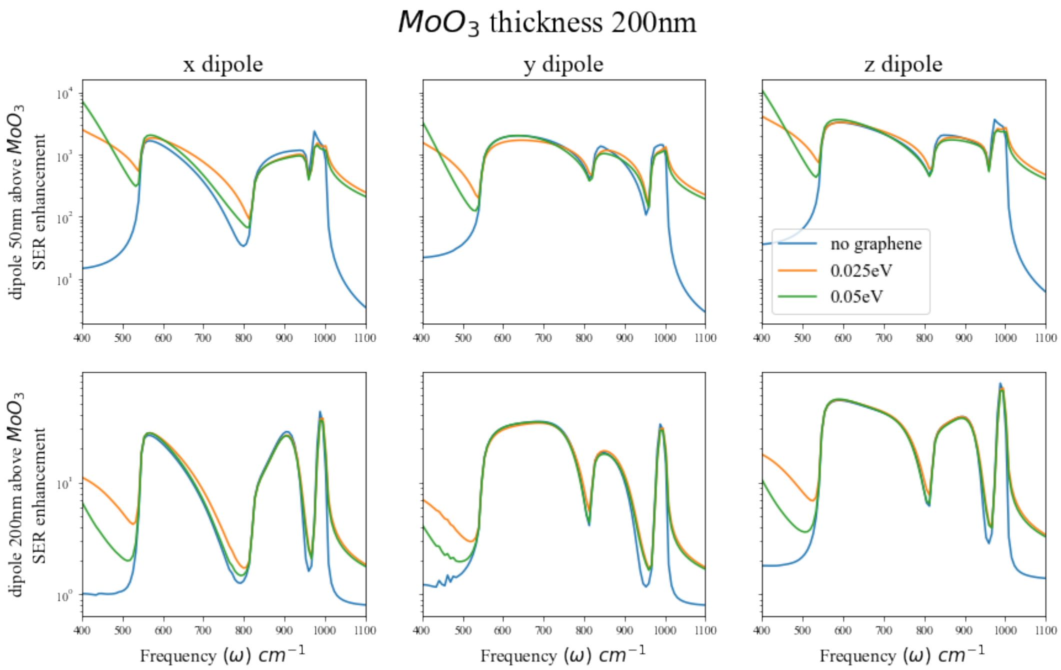

FIG. S10. Anisotropic SERs from a 200nm thin film of α-MoO_3: (a)-(c) represent SERs of $x-$, $y-$ and $z-$ polarized electric dipole, respectively, at $z_\circ = 50$nm for different μ values. (d)-(f) represent SERs of $x-$, $y-$ and $z-$ polarized electric dipole, respectively, at $z_\circ = 200$nm for different μ values.

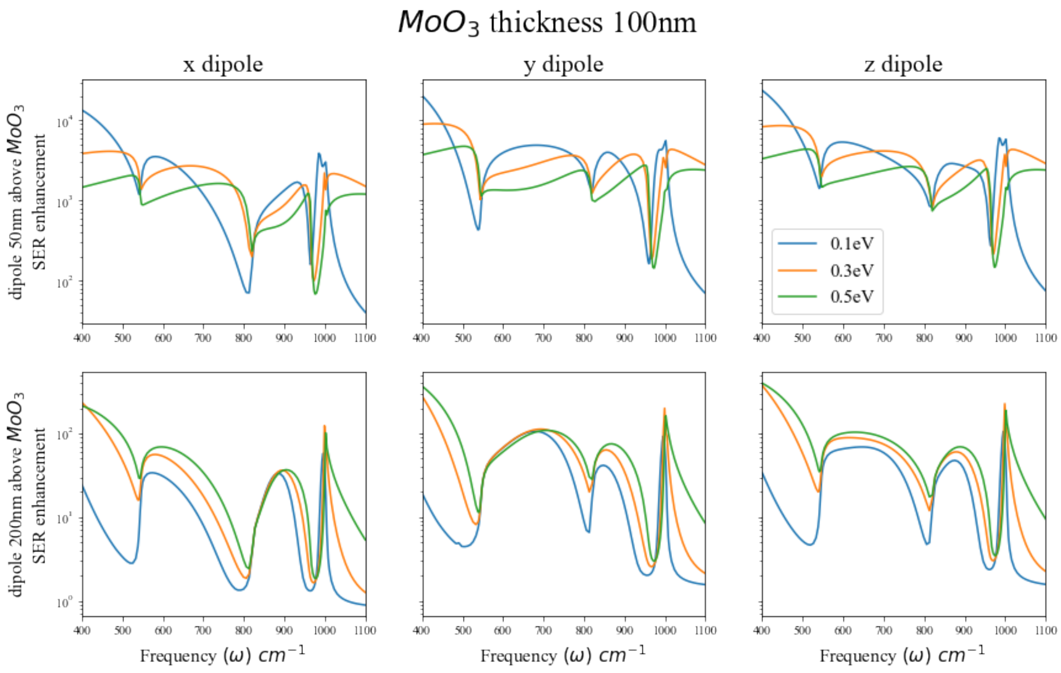

FIG. S11. Anisotropic SERs from a 100nm thin film of α-MoO$_3$: (a)-(c) represent SERs of $x-$, $y-$ and $z-$ polarized electric dipole, respectively, at $z_\circ = 50$nm for $\mu = 0.1eV$, $0.3eV$, and $0.5eV$. (d)-(f) represent SERs of $x-$, $y-$ and $z-$ polarized electric dipole, respectively, at $z_\circ = 200$nm for for $\mu = 0.1eV$, $0.3eV$, and $0.5eV$.

C. Anisotropic SERs of α-MoO$_3$ and graphene heterostructure forming HPPPs

For $\mu = 0.1eV$, $0.3eV$, and $0.5eV$, the formation of HPPPs is substantiated by dispersion curves shown in Figs.S2 and S3. Anisotropic SERs spectra from 50nm thin film of α-MoO$_3$ have been shown in Fig.4 of main manuscript. Here, we present effect of thickness of α-MoO$_3$ on anisotropic SERs for $\mu = 0.1eV$, $0.3eV$, and $0.5eV$. Thicknesses of α-MoO$_3$ are considered 100nm and 200nm and their anisotropic SERs are shown in Figs.S11 and S12 respectively. Order of anisotropic SERs spectra, at $z_\circ = 50$nm and 200nm, for both the thicknesses remains almost identical with spectra observed for 50nm thin film of α-MoO$_3$ shown in Fig. 4 of main text. The variation of SERs spectra inside the RB spectral region is due to variation in dispersion of HPPP modes via chemical potential. The line-width and oscillator strength of spectral dips decreases with increasing thickness due to, as discussed above, change in the phononic density of states with thickness of α-MoO$_3$.

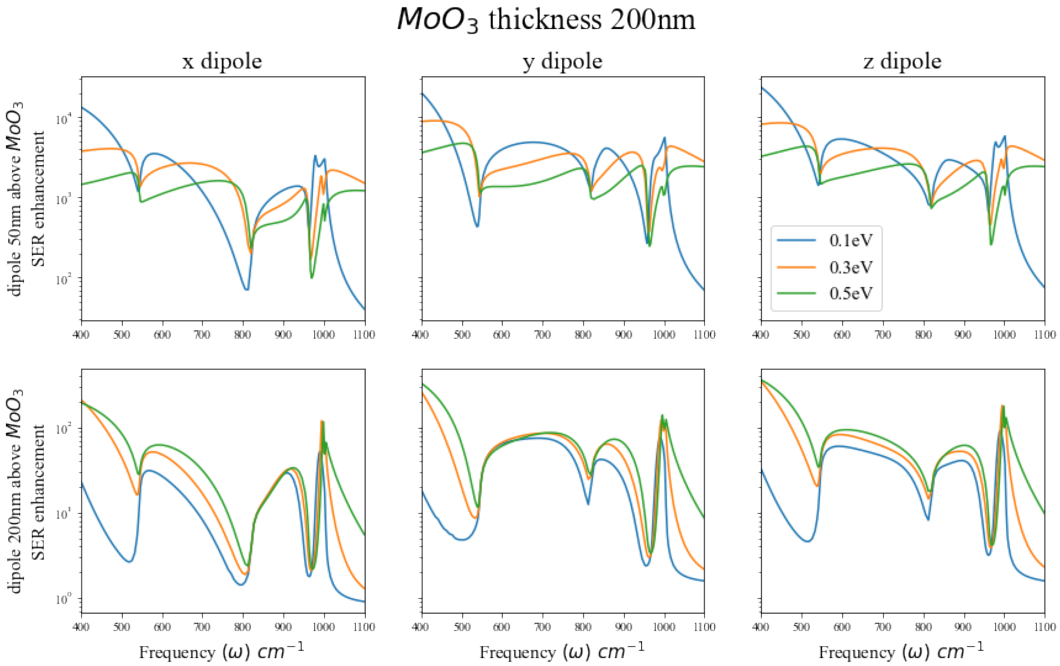

FIG. S12. Anisotropic SERs from a 200nm thin film of α-MoO_3: (a)-(c) represent SERs of $x-$, $y-$ and $z-$ polarized electric dipole, respectively, at $z_\circ = 50$nm for $\mu = 0.1eV$, $0.3eV$, and $0.5eV$. (d)-(f) represent SERs of $x-$, $y-$ and $z-$ polarized electric dipole, respectively, at $z_\circ = 200$nm for for $\mu = 0.1eV$, $0.3eV$, and $0.5eV$.

S5. SPONTANEOUS EMISSION RATES FOR AIR/GRAPHENE/SI HETEROSTRUCTURE

Spontaneous emission rates for a dipole placed 50nm and 200nm above an air/graphene/Si heterostructure (without MoO_3) is presented in figure S13. We can observe that the spontaneous emission rate goes on decreasing for $\mu = 0.1$eV as we increase the frequency. This is because as we increase the frequency, the SPP dispersion moves to larger wavevector which cannot interact effectively with the dipoles at the given heights. For 0.3eV and 0.5eV, the SPP mode exists at a lower wavevector as compared to 0.1eV. Thus, the Purcell factor is nearly constant for $z_0 = 50$nm, but not for $z_0 = 200$nm where the accessible wavevectors are smaller than 50nm due to exponentially decaying fields. $z_0 = 200$nm follows the same decaying trend as 0.1eV as described before. Since MoO_3 is not present in this system, the Purcell factor of all horizontal directions is identical.

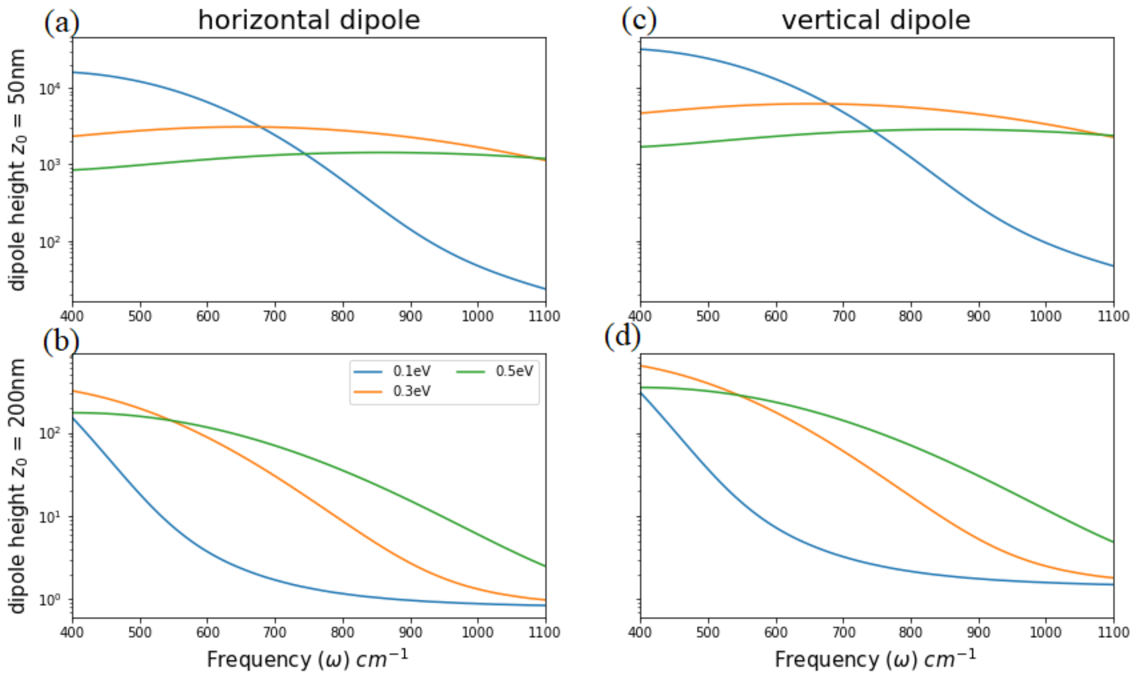

FIG. S13. SER enhancement for a dipole placed above an air/graphene/Si substrate. SERs for horizontal dipoles (parallel to the substrate) are plotted in (a,b) and for vertical dipoles (perpendicular to the substrate) are plotted in (c,d). Also, (a),(c) have the height of the dipole above the substrate as 50nm whereas (b),(d) have the dipole placed 200nm above the substrate.

S6. CONVERGENCE TESTS

From the Green's tensor method presented in the methods section of the main text, after a changing to polar coordinates and then changing the integration variable to k_z (z wavevector in air), we can obtain the following expressions for spontaneous emission enhancement in x,y and z oriented dipoles -

$$\frac{P_x}{P_{x0}} = 1 + \frac{3}{4\pi} Im \Bigg(\int_0^1 \int_0^{2\pi} (ir_{ss}\sin^2\phi - ir_{pp}k_z'^2\cos^2\phi + ik_z'(r_{ps}-r_{sp})\sin\phi\cos\phi) e^{2ik_z'k_0z_0} d\phi dk_z' + \int_0^\infty \int_0^{2\pi} (r_{ss}\sin^2\phi + r_{pp}k_z''^2\cos^2\phi + ik_z''(r_{ps}-r_{sp})\sin\phi\cos\phi) e^{-2k_z''k_0z_0} d\phi dk_z'' \Bigg)$$

$$\frac{P_y}{P_{y0}} = 1 + \frac{3}{4\pi} Im \Bigg(\int_0^1 \int_0^{2\pi} (ir_{ss}\cos^2\phi - ir_{pp}k_z'^2\sin^2\phi - ik_z'(r_{ps}-r_{sp})\sin\phi\cos\phi) e^{2ik_z'k_0z_0} d\phi dk_z' + \int_0^\infty \int_0^{2\pi} (r_{ss}\cos^2\phi + r_{pp}k_z''^2\sin^2\phi - ik_z''(r_{ps}-r_{sp})\sin\phi\cos\phi) e^{-2k_z''k_0z_0} d\phi dk_z'' \Bigg)$$

$$\frac{P_z}{P_{z0}} = 1 + \frac{3}{4\pi} Im \Bigg(\int_0^1 \int_0^{2\pi} (ir_{pp}(1-k_z'^2)) e^{2ik_z'k_0z_0} d\phi dk_z' + \int_0^\infty \int_0^{2\pi} (r_{pp}(k_z''^2+1)) e^{-2k_z''k_0z_0} d\phi dk_z'' \Bigg)$$

where, in each case, the first integral represents an integral over propagating modes, with $k_z' = \frac{k_z}{k_0}$ and the second integral is over evanescent modes with $k_z'' = \frac{k_z}{ik_0}$.

For generating the Purcell spectra and degree of anisotroy plots presented in the main text, we used a 2D trapezoidal rule over a sufficiently large finite box to estimate the second integral over evanescent modes. There are two possible sources of error here - the finite upper bound and the fineness of the mesh. Figure S14 presents the plots which support that our estimate is well converged in both these aspects. For this, we have performed the following numerical tests -

1. Finer mesh - The number of mesh nodes in both k_z" and ϕ were doubled.

2. Half the upper bound - The upper bound on k_z" was taken as $12 \times 10^6/(z_\circ w)$ where $z_\circ$ is in nm and w is in cm^{-1} for evaluating the integral in the plots in the main manuscript. This specific choice was based on the argument of the decaying exponential. Here, we calculate the integral with the upper bound taken as half of the above expression, i.e. $6 \times 10^6/(z_\circ w)$

3. $3/4^{\text{th}}$ upper bound - The test here is similar to the test of half the upper bound, but with $9 \times 10^6/(z_\circ w)$ taken as upper bound. The purpose of this test is to contrast with

the half upper bound, and see that the error has gone down significantly, which shows that we have reached a sufficiently high upper bound.

Since the contribution of the evanescent modes is larger in the case of 50nm, the results of these 3 tests for dipole height $z_\circ = 50nm$ are most critical and these are presented in figure S14. As we can see from it, the percent error introduced in our results from either source are relatively small.

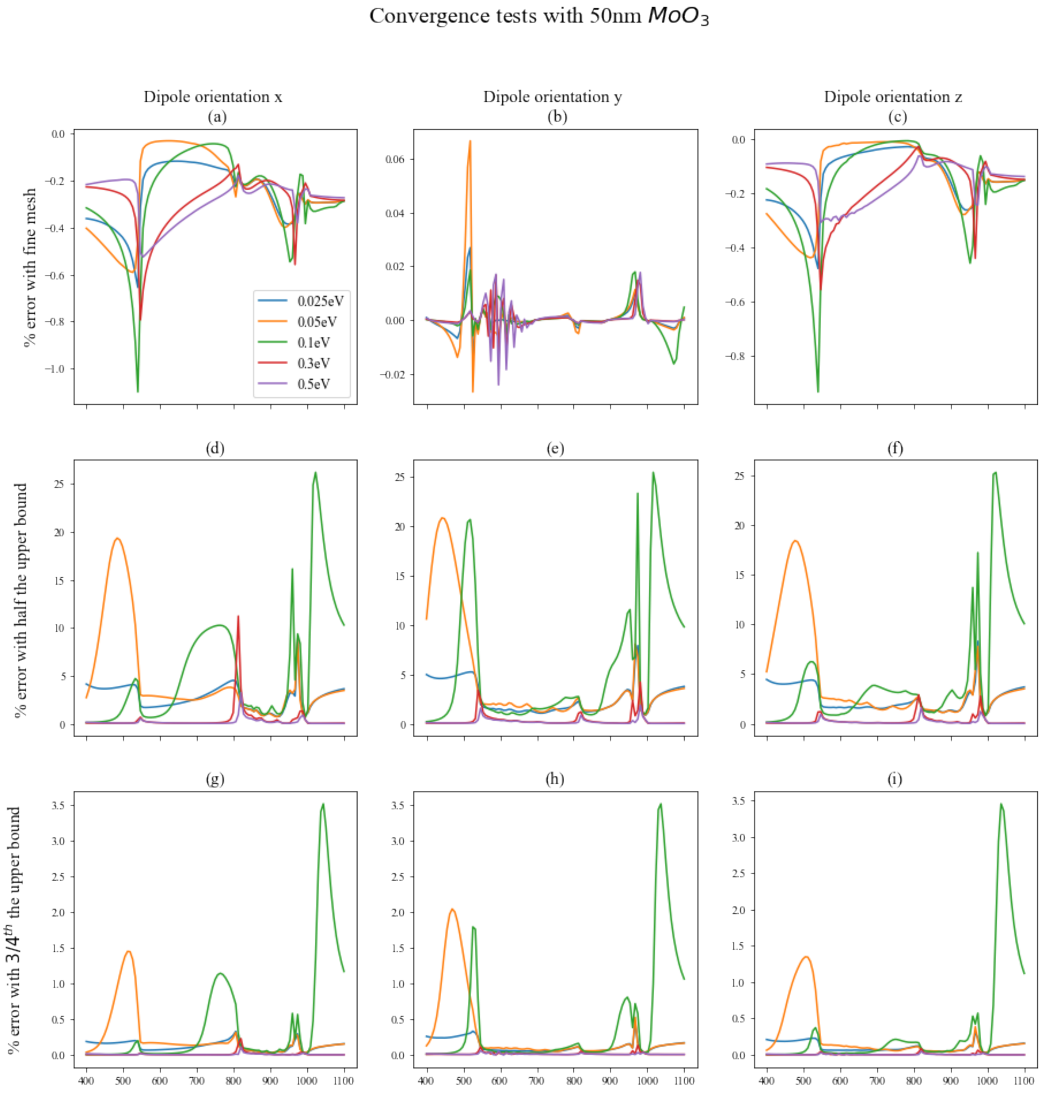

FIG. S14. Convergence tests performed for 50nm thickness of MoO_3. The three rows correspond to mesh refinement, half upper bound and $3/4^{\rm th}$ upper bound tests described in the text, respectively. Vertical axes in percent error units.

S7. DISCUSSION OF ANALYTICAL APPROXIMATE EXPRESSION FOR DISPERSION

In the main text, we propose the following approximate expression for the dispersion relation in the limit of $k_\rho \gg k_0$ -

$$\frac{k_\rho}{k_0}(\omega) = \frac{\psi}{k_0 d}\left(\tan^{-1}\left(\frac{\psi(\epsilon_1 + i\left(\frac{k_\rho}{k_0}\right)\sigma Z_0)}{\epsilon_z}\right) + \tan^{-1}\left(\frac{\epsilon_3\psi}{\epsilon_z}\right) + n\pi\right) \tag{S1}$$

Where $\psi = i\sqrt{\epsilon_z/(\epsilon_x\cos^2\phi + \epsilon_y\sin^2\phi)}$.The reason behind this is as follows - the Fresnel equation for propagation of electromagnetic waves in biaxial media is written as [S1] -

$$-q_z^2(-q_z^2\epsilon_z + \epsilon_z(\epsilon_x + \epsilon_y) - q_x^2(\epsilon_x + \epsilon_z) - q_y^2(\epsilon_y + \epsilon_z)) + (\epsilon_z - q_x^2 - q_y^2)(\epsilon_x\epsilon_y - q_x^2\epsilon_x - q_y^2\epsilon_y) = 0$$

Where $q_i = \frac{k_i}{k_0}$ $(i = x, y, z)$ are the normalised wave vectors(note that the convention used for q_z here is slightly different from [S1]). If we consider the quantities $q_\rho = q_x^2 + q_y^2 = \frac{k_\rho}{k_0}$ and $\epsilon_\rho = \epsilon_x\cos^2\phi + \epsilon_y\sin^2\phi$ so that $q_x^2\epsilon_x + q_y^2\epsilon_y = q_\rho^2\epsilon_\rho$, the above equation simplifies to

$$-q_z^2(-q_z^2\epsilon_z + \epsilon_z(\epsilon_x + \epsilon_y) - q_\rho^2(\epsilon_\rho + \epsilon_z)) + (\epsilon_z - q_\rho^2)(\epsilon_x\epsilon_y - q_\rho^2\epsilon_\rho) = 0$$

then neglecting all ϵ terms in comparison to q terms above (which is valid if $k_\rho \gg k_0$, this is the so-called quasistatic approximation), the solution to the above Fresnel equation looks like $q_\rho^2 \approx -q_z^2$ or $\epsilon_\rho q_\rho^2 \approx -\epsilon_z q_z^2$. We can treat propagating modes in the biaxial medium as 's-like' and 'p-like' in the quasistatic limit.

The rest of the analysis is similar to [S2], and we arrive at a similar looking equation that describes the dispersion modes. Note that the anisotropy and ϕ-dependence comes in from the expression for ϵ_ρ, which depends on ϕ. One important remark about the integer n in the expression, is that we should choose $n = 0, 1, 2, 3\ldots$ outside RB3 and $n = 0, -1, -2, -3\ldots$ within RB3. This is equivalent to choosing the sign of ψ, as discussed in [S2].

We now present a comparison of this approximation to full wave calculations presented in all sections so far. In figure S15 we plot $\left(-\sum_{n=0}^{4} log\left|\frac{k_\rho}{k_0} - f\left(\frac{k_\rho}{k_0}, \omega, \pm n\right)\right|\right)$ where $f\left(\frac{k_\rho}{k_0}, \omega, \pm n\right)$ is the right hand side of equation S1 and the sign of $\pm n$ is chosen as discussed above. This quantity would have a larger value at $\left(\frac{k_\rho}{k_0}, \omega\right)$ combinations where the analytical expression holds and thus highlight the analytical dispersion contours. We can see that these compare favourably to figure 2 of the main text, which shows dispersion relations obtained from full wave calculations.

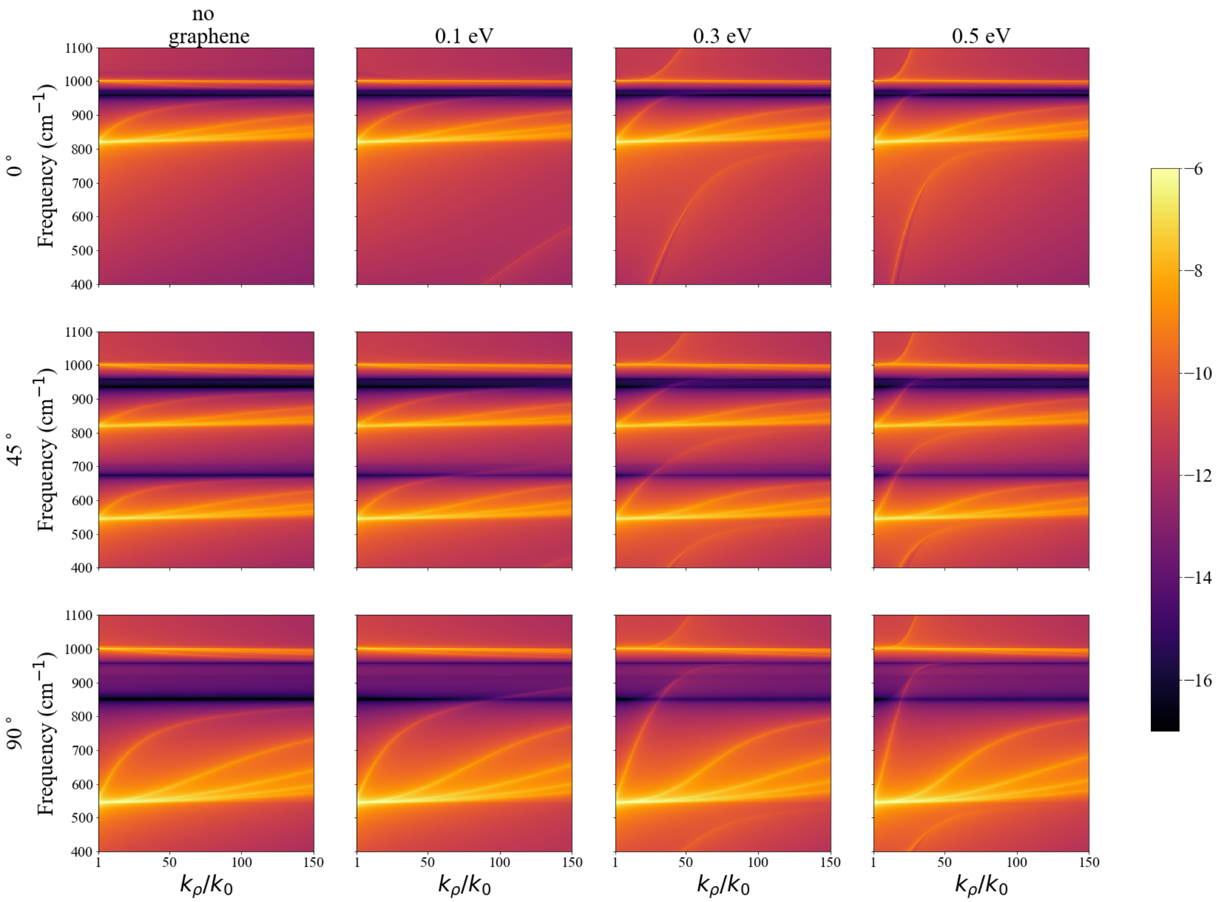

FIG. S15. $\left(-\sum_{n=0}^{4} log\left|\frac{k_\rho}{k_0} - f\left(\frac{k_\rho}{k_0}, \omega, \pm n\right)\right|\right)$ as a function of $\left(\frac{k_\rho}{k_0}, \omega\right)$. The bright lines here indicate the curves along which the analytic condition is closely met (for the first 5 mode orders), and we can see that they compare favourably to figure 2 of the main text (showing full wave calculation dispersion contours)

[S1] G. Álvarez-Pérez, K. V. Voronin, V. S. Volkov, P. Alonso-González, and A. Y. Nikitin, Phys. Rev. B **100**, 235408 (2019).

[S2] A. Kumar, T. Low, K. H. Fung, P. Avouris, and N. X. Fang, Nano Letters **15**, 3172 (2015)
